# Performance Comparison among the Major Healthcare Financing Systems in Six Cities of the Pearl River Delta Region, Mainland China

**DOI:** 10.1371/journal.pone.0046309

**Published:** 2012-09-28

**Authors:** Martin C. S. Wong, Harry H. X. Wang, Samuel Y. S. Wong, Xiaolin Wei, Nan Yang, Zhenzhen Zhang, Haitao Li, Yang Gao, Donald K. T. Li, JinLing Tang, Jiaji Wang, Sian M. Griffiths

**Affiliations:** 1 School of Public Health and Primary Care, Faculty of Medicine, Chinese University of Hong Kong, Hong Kong, Hong Kong, Special Administrative Region, People’s Republic of China; 2 Bauhinia Foundation Research Centre, Hong Kong, Hong Kong, Special Administrative Region, People’s Republic of China; 3 School of Public Health, Guangzhou Medical University, Guangzhou, People’s Republic of China; UNAIDS, Switzerland

## Abstract

**Background:**

The healthcare system of mainland China is undergoing drastic reform and the optimal models for healthcare financing for provision of primary care will need to be identified. This study compared the performance indicators of the community health centres (CHCs) under different healthcare financing systems in the six cities of the Pearl River Delta region.

**Methods:**

Approximately 300 hypertensive patients were randomly recruited from the computerized chronic disease management records provided by one CHC in each of the six cities in 2011 using a multi-stage cluster random sampling method. The major outcome measures included the treatment rate of hypertension, defined as prescription of ≥ one antihypertensive agent; and the control rate of hypertension, defined as systolic blood pressure levels <140 mmHg and diastolic blood pressure levels <90 mmHg in patients without diabetes mellitus, or <130/80 mmHg among patients with concomitant diabetes. Binary logistic regression analyses were conducted with these two measures as outcome variables, respectively, controlling for patients’ socio-demographic variables. The financing system (Hospital- vs. Government- vs. private-funded) was the independent variable tested for association with the outcomes.

**Results:**

From 1,830 patients with an average age of 65.9 years (SD 12.8), the overall treatment and control rates were 75.4% and 20.2%, respectively. When compared with hospital-funded CHCs, patients seen in the Government-funded (adjusted odds ratio [AOR] 0.462, 95% C.I. 0.325–0.656) and private-funded CHCs (AOR 0.031, 95% C.I. 0.019–0.052) were significantly less likely to be prescribed antihypertensive medication. However, the Government-funded CHC was more likely to have optimal BP control (AOR 1.628, 95% C.I. 1.157–2.291) whilst the privately-funded CHC was less likely to achieve BP control (AOR 0.146, 95% C.I. 0.069–0.310), irrespective of whether antihypertensive drugs were prescribed.

**Conclusions:**

Privately-funded CHCs had the lowest rates of BP treatment and control due to a variety of potential factors as discussed.

## Introduction

Primary care was defined by the WHO as the “the first level of contact of individuals, the family and the community with the national health system, bringing health care as close as possible to where people live and work” [1]. A substantial body of evidence around the world has suggested that primary care, in contrast to specialty care, is associated with lower morbidity and mortality as well as more equitable distribution of health within and across populations [2]. Health systems with more primary care orientation could lead to better health outcomes, induce lower healthcare costs, and enhance patient satisfaction [3], [4]. The WHO has therefore emphasized the urgency of enhancing primary care [5], and it is widely recognized that healthcare reforms need to begin with primary care [2]. From the global perspective, there is an international trend of health policy orientation towards developing primary care as a priority of health reforms to improve health outcomes [6]. These include European countries, Brazil, Australia and Asia-Pacific countries [7]–[10].

In early 2009, the Chinese government initiated a comprehensive healthcare reform [11] which stands on four key pillars, including healthcare financing, care delivery, drug supply, and hospital reforms. Initiatives to systematically strengthen community-based primary care services are being put at the top of the agenda to underpin the care delivery pillar in order to provide universally accessible services at affordable prices to those in need. The set up of community health centres (CHCs) in nearly every neighbourhood in urban areas has been particularly prominent as a new initiative to address a broad range of patients’ healthcare needs by providing community health services.

CHCs in each city of the Pearl River Delta (PRD) region of China were operated by one of the three distinct healthcare financing systems, namely hospital-, Government- and private-funding, defined by the source of budgeting and ownership. The PRD refers to the network of 6 cities in the Guangdong province. It has a total population of 43.1 million in 2010 [12], and is the leading economic region and main hub of China’s economic growth [13]. This region enables researchers a unique opportunity to study the strengths and weaknesses of different healthcare delivery models. However, there is a scarcity of studies which compared the performance of primary care delivered by these different models. Identifying the financing model that leads to better health outcomes could inform policy-makers on the future planning and implementation of health delivery strategies.

The government plays a dominant role in financing and ownership in the health sector in China. Government-owned CHCs are established by and subject to the local government as non-profit healthcare facilities which are detached from hospitals [14]. Local government operates and manages the CHCs exclusively, whereas the local health bureau provides professional guidance, industrial monitoring and assessment to guarantee the quality of service provision. Hospital-owned CHCs have a tight relationship with various types of non-private hospitals, and the host public hospitals are responsible for daily operation and administration of the CHCs, with the provision of healthcare professionals, salary, medical equipments, technique support, funding resource managed in an integrative approach [15], [16]. Privately-owned CHCs are established, owned, and operated by private/social investors along with local government and hospitals to provide comparable primary care services [17].

The management of hypertension, one of the most commonly encountered chronic conditions in primary care, has been widely accepted as a recognized proxy measure of clinical performance [14]–[15]. The treatment and blood pressure (BP) control rates among hypertensive patients are valid across localities and countries, and have been used extensively as outcome indicators for assessing the quality of healthcare systems [16]. The objective of this study is to compare the performance of the three major healthcare financing systems in the PRD region using management of hypertension as an outcome measure. We tested the *a priori* hypothesis that there exist differences in the performance among these three financing models.

## Materials and Methods

### Ethics Statement

This study was approved by the Survey and Behavioral Research Ethics Committee of the Faculty of Medicine, Chinese University of Hong Kong.

### Data Source & Sampling Methodology

The computerized chronic disease management records were used to retrieve data on the management of patients with hypertension by primary care providers in the CHCs of the cities under study. The data for the outcome part were retrieved from a previous “Community-wide Household Health Assessment (CHHA) Project”, which collected healthcare records of adult residents who hold the “Hukou” registry from 5% of the general population in the six cities of Pearl River Delta in 2010. “Hukou” registry refers to those who have relatively more permanent residence, and is a household registration related to the migration control and resource distribution under China’s residential policy. Its registration entitled people the access to basic social services, old-age welfare, subsidized housing, more opportunities for employment, and free public education in their specific registered area. Multistage cluster random sampling method was adopted in retrieving the data in the current study, as this is more feasible and easier to coordinate. In each city, one district was randomly selected as the “Primary Sampling Units” (PSU). One neighborhood was selected by simple random sampling as the “Secondary Sampling Units” (SSU) within the district. One CHC was selected by simple random sampling as the “Tertiary Sampling Units” (TSU) within the neighborhood (**Table 1**). From all the eligible hypertensive patients in each selected CHC, approximately 300 patients were included in the study by simple random sampling with one patient being counted as one unit of randomization. For each patient, a unique identifier “1, 2, 3…etc.” was assigned according to their citizen card number, which served the purpose to anonymize individual information and protected patients from being identified.

**Table 1:** The characteristics of the six cities under study.

City	A	B	C	D*	E	F
**Type**	Provincial Capital	Prefectural level city	Prefectural level city	Special EconomicZone	Prefectural level city	Special Economic Zone
**Primary Care Providers**	Hospital- ownedCHC	Hospital- owned CHC	Hospital- owned CHC	Hospital- owned CHC	Government- owned CHC	Privately- owned CHC
**No. of CHCs**	118	45	28	68	32	10
**No. of districts**	11	5	28	8	32	10
**No. of PSU**	1	1	1	1	1	1
**No. of SSU**	1	1	1	1	1	1
**No. of TSU**	1	1	1	1	1	1
**No. of Total Subjects**	305	301	302	309	300	303

* The number of CHCs in city D is counted according to the number of host hospitals. PSU: Primary Sampling Units; SSU: Secondary Sampling Units; TSU: Tertiary Sampling Units; CHC: Community Health Centre.

### Study Participants

The study period was January to October, 2010. The inclusion criteria used in selecting subjects for this study include 1). patients who had identifiable primary care providers and who were previously service users in the selected CHCs; 2). those who received a diagnosis of hypertension by the primary care physicians in the selected CHCs; and 3). those who have been living in the neighborhood covered by the selected CHCs for at least 1 year, in order to include subjects with adequately long periods for continuity of clinical care.

### Outcome Measures and Covariates

The primary outcomes include 1). treatment of hypertension, defined as the prescription of at least one medication within the major antihypertensive drug classes in the study period of 2010; and 2). control of hypertension, defined as an average of two blood pressure levels measured in the CHCs being <140 mmHg (systolic) and <90 mmHg (diastolic) in patients without diabetes. For each patient, we used the average of the first two BP values in the calendar year 2010. In the presence of diabetes, levels of <130 mmHg (systolic) and <80 mmHg (diastolic) were adopted according to the recommendations of the Joint National Committee 7^th^ report [17].

The covariates include patients’ age, gender, occupation (employed vs. unemployed vs. retired or others), educational status (primary or lower vs. secondary vs. tertiary or above), presence of medical insurance coverage, marital status (single vs. married vs. divorced/separated/widowed) and body mass index. The insurance system and the amount of insurance coverage on fees from clinical examination, medical treatment, and medication costs were in general of similar magnitude when compared among CHCs in these cities [18]; whilst there are some conditions that could influence the proportion of insurance reimbursement for each individual, including the severity of the medical condition, insurance category of the drugs, whether the healthcare facilities where healthcare expenditures occurred are enlisted in the insurance, employment status and hierarchy, etc. [19]. The predictor variable tested for association with the two outcome variables was the financing type of the CHCs (Government vs. hospital vs. private-funding). It was hypothesized that there exists difference in the performance indicators among the different CHCs funded under the three distinct financing models.

### Statistical Analysis

The Statistical Package for Social Sciences (SPSS) version 15.0 was used for all data entry and analysis. The demographic and clinical characteristics of patients under each CHC were compared by χ^2^ tests of heterogeneity and Student’s t-tests for categorical and continuous variables, respectively. The treatment and control rates of the six CHCs were expressed in proportions and compared. Two separate binary logistic regression models were constructed with treatment and control of hypertension as outcome variables, respectively. An additional regression model was conducted with presence of antihypertensive treatment as a covariate and BP control as an outcome variable. The above analysis was run again by merging the four hospital-managed CHCs (in cities A to D) as one group under “hospital-funding”. To evaluate the independent association between the outcome variables and the financing system, all covariates were entered into the regression analyses. P values <0.05 were regarded as statistically significant.

## Results

### Patient Characteristics

The demographic characteristics of the patients were shown in **Table 2**. The average age of the study subjects was 65.9 years (SD 12.8). Male patients consist of 48.6%. Only 21.6% of the participants were employed full-time. The majority received primary education or below (51.8%). Most were married (93.8%) and under medical insurance (62.4%). Approximately 13.3% had concomitant diabetes mellitus at the time of the survey, and the mean duration of hypertension was 5.7 years (SD 6.3). Their average systolic and diastolic BP levels were 147.6 and 88.4 mmHg, respectively. They had an average BMI of 24.1 kg/m^2^. There were significant heterogeneity among the six cities according to all the demographic and clinical variables (all p<0.001).

**Table 2:** Patient Characteristics (N = 1,830).

	Hospital Funded				Govt funded	Private funded	All patients	p**
	A	B	C	D	E	F		
	(n = 309)	(n = 302)	(n = 305)	(n = 303)	(n = 310)	(n = 301)		
**Age** *	69.8	67.4	66.9	69.3	65.3	56.9	65.9	<0.001
**(years)**	−10.4	−10.7	−10.2	−12.8	−14.1	−13.4	−12.8	
**Gender**								
Male	138 (44.7)	135 (44.7)	152 (49.8)	130 (42.9)	143 (46.1)	191 (63.5)	889 (48.6)	<0.001
Female	171 (55.3)	167 (55.3)	153 (50.2)	173 (57.1)	167 (53.9)	110 (36.5)	941 (51.4)	
**Occupation**								
Employed	37 (12.5)	99 (32.8)	24(7.9)	47 (38.2)	49(15.8)	72(38.9)	328 (21.6)	<0.001
Unemployed	71 (23.9)	97 (32.1)	203 (66.8)	23 (18.7)	165 (53.2)	49(26.5)	608 (40.0)	
Retired/others	189 (63.6)	106 (35.1)	77 (25.3)	53 (43.1)	96(31.0)	64(34.6)	585 (38.5)	
**Educational status**								
≤Primary	82 (27.7)	210 (69.8)	204 (67.1)	23 (18.7)	230 (76.2)	33(17.8)	782 (51.8)	<0.001
Secondary	148 (50.0)	75 (24.9)	84 (27.6)	69 (56.1)	71(23.5)	111 (60.0)	558 (36.9)	
≥ Tertiary	66 (22.3)	16(5.3)	16(5.3)	31 (25.2)	1(0.3)	41(22.2)	171 (11.3)	
**Marital status**								
Single	2 (0.7)	0 (0.0)	1 (0.3)	0 (0.0)	4 (1.3)	8 (4.3)	15 (1.0)	<0.001
Married	292 (98.3)	294 (97.4)	298 (98.0)	123 (100.0)	234 (78.3)	176 (95.1)	1417 (93.8)	
Divorced	1 (0.3)	0 (0.0)	0 (0.0)	0 (0.0)	1 (0.3)	0 (0.0)	2 (0.1)	
Widowed	2 (0.7)	8 (2.6)	5 (1.6)	0 (0.0)	60 (20.1)	1 (0.5)	76 (5.0)	
**Medical insurance**								
No	52 (16.8)	56 (18.5)	148 (48.5)	234 (77.2)	74 (23.9)	124 (41.2)	688 (37.6)	<0.001
Yes	257 (83.2)	246 (81.5)	157 (51.5)	69 (22.8)	236 (76.1)	177 (58.8)	1142 (62.4)	
**Presence of diabetes**								
No	260 (84.1)	273 (90.4)	243 (79.7)	280 (92.4)	274 (88.4)	257 (85.4)	1587 (86.7)	<0.001
Yes	49 (15.9)	29(9.6)	62 (20.3)	23(7.6)	36(11.6)	44(14.6)	243 (13.3)	
**Duration of hypertension** *	7.7	5.6	6.2	5.4	5.3	4.6	5.7	<0.001
(mean in years)	−8.1	−6.3	−6.3	−7.0	−4.8	−4.1	−6.3	
**Mean SBP** *	148.6	149.1	147.1	145.7	143.0	151.9	147.6	<0.001
(mmHg)	−15.3	−14.6	−15.7	−14.0	−16.9	−12.1	−15.1	
**Mean DBP** *	87.3	87.0	86.3	86.6	86.0	97.6	88.4	<0.001
(mmHg)	−11.3	−10.0	−10.4	−9.5	−11.3	−10.3	−11.2	
**BMI** *	24.6	24.0	24.3	23.2	24.9	23.6	24.1	<0.001
(m/kg^2^)	−3.4	−3.5	−3.3	−2.8	−4.1	−2.8	−3.4	

SBP: Systolic Blood Pressure; DBP: Diastolic Blood Pressure; BMI: Body Mass Index. A to F represent selected community health centres in the six cities of the Pearl River Delta region.

* Continuous variables;

** chi-square test for categorical variables and Student’s t-tests for continuous variable. The figures in the brackets represent standard deviations and percentages across columns for continuous and categorical variables, respectively.

### Treatment and Control Rates

The overall treatment and control rates of all patients were 75.4% and 20.2%, respectively (**Table 3**). Among patients on antihypertensive drug treatment, the BP control rate was 23.8%. Hospital-funded CHCs had the highest treatment (range: 83.1%–92.1%) rates, followed by Government (70.3%) and privately-funded CHCs (29.9%). The BP control rates were highest among the Government-funded CHC (25.8%), followed by hospital-funded CHCs (22.7%, range 20.1%–26.7%) and the privately funded CHC (8.9%). This is similarly found among patients on antihypertensive treatment, where the control rates were higher in the Government-funded CHC (33.0%) than hospital-funded (23.2%, range 20.1%–28.9%) and private-funded CHC (8.9%).

**Table 3:** Treatment and Control Rates of hypertension in the Community Health Centres (CHCs) in the six cities of the Guangdong Province.

City	Type	Number of patients	Treatment Rate	Control Rate (all patients)	Control Rate (among patients with treatment)
A	Hospital-funded	309	84.8% (262/309)	20.1% (62/309)	20.1% (51/262)
B	Hospital-funded	302	83.1% (251/302)	21.5% (65/302)	20.7% (52/251)
C	Hospital-funded	305	92.1% (281/305)	22.6% (69/305)	23.1% (65/281)
D	Hospital-funded	303	91.4% (277/303)	26.7% (81/303)	28.9% (80/277)
E	Government-funded	310	70.3% (218/310)	25.8% (80/310)	33.0% (72/218)
F	Private-funded	301	29.9% (90/301)	4.3% (13/301)	8.9% (8/90)
Total		1,830	75.4% (1379/1830)	20.2% (370/1,830)	23.8% (328/1,379)

A to F represent selected community health centres in the six cities of the Pearl River Delta region. Treatment was defined as the prescription of at least one antihypertensive agent; control was defined as systolic blood pressure <140 mmHg and diastolic blood pressure <90 mmHg; or <130 mmHg and <80 mmHg, respectively in patients with diabetes mellitus.

### Factors Associated with Antihypertensive Treatment and Optimal BP Control

**Table 4** shows the association between treatment and control of hypertension, respectively, with the various CHCs when the potential confounders were controlled. It was found that when compared with city A, patients seen in city C (aOR 2.865, 95% C.I. 1.612–5.095, p<0.001) were more likely to receive an antihypertensive medication, whereas patients in city E (adjusted odds ratio [aOR] 0.627, 95% C.I. 0.393–1.000, p = 0.050) and city F (aOR 0.038, 95% C.I. 0.021–0.068, p<0.001) were less likely to be offered antihypertensive treatments. In addition, CHCs in city C (aOR 1.641, 95% C.I. 1.052–2.560, p = 0.029) and city E was significantly more likely (aOR 2.231, 95% C.I. 1.406–3.538, p = 0.001) while city F significantly less likely (aOR 0.184, 95% C.I. 0.083–0.411, p<0.001) to have optimal blood pressure control among hypertensive patients. When the regression analysis was re-conducted with antihypertensive treatment included as a covariate, similar findings were obtained. Patients in city E were more likely (aOR 2.401, 95% C.I. 1.502–3.838, p<0.001) and in city F less likely to achieve optimal BP control (aOR 0.296, 95% C.I. 0.127–0.691, p = 0.005) (**Table 5**).

**Table 4:** Factors associated with antihypertensive drug treatment and optimal control of blood pressure (with individual city as a covariate).

	Treatment by antihypertensive drug (R^2^ = 0.397)				Optimal blood pressure control (R^2^ = 0.082)				Optimal blood pressure control with antihypertensive treatment as a covariate (R^2^ = 0.093)			
	n	%	Adjusted odds ratio (95% C.I.)	p	n	%	Adjusted odds ratio (95% C.I.)	p	n	%	Adjusted odds ratio(95% C.I.)	p
**Age (years)**			**1.035 (1.022–1.049)**	**<0.001**			**1.013 (1.000–1.026)**	**0.043**			1.011 (0.998–1.024)	0.105
**Gender**												
Male	633	71.2	Ref.	0.998	172	19.3	Ref.	0.309	172	19.3	Ref.	0.306
Female	746	79.3	1.000 (0.741–1.348)		198	21.0	1.150 (0.878–1.506)		198	21.0	1.152 (0.879–1.510)	
**Occupation**												
Employed	208	63.4	Ref.		63	19.2	Ref.		63	19.2	Ref.	
unemployed	451	74.2	0.784 (0.522–1.177)	0.240	123	20.2	1.060 (0.713–1.577)	0.773	123	20.2	1.077 (0.722–1.608)	0.716
Retired/in school/others	460	78.6	1.191 (0.784–1.810)	0.412	124	21.2	1.010 (0.684–1.493)	0.960	124	21.2	1.007 (0.678–1.493)	0.974
**Educational status**												
Primary or lower	626	80.1	Ref.		156	19.9	Ref.		156	19.9	Ref.	
Secondary	368	65.9	0.875 (0.617–1.239)	0.450	106	19.0	**1.475 (1.058–2.058)**	**0.022**	106	19.0	**1.479 (1.057–2.068)**	**0.022**
Tertiary or above	121	70.8	1.602 (0.884–2.905)	0.120	47	27.5	**3.101 (1.906–5.044)**	**<0.001**	47	27.5	**2.976 (1.824–4.856)**	**<0.001**
**Medical insurance**												
Absence	583	84.7	Ref.		135	19.6	Ref.		135	19.6	Ref.	
Presence	796	69.7	0.822 (0.564–1.198)	0.307	235	20.6	1.258 (0.912–1.735)	0.162	235	20.6	1.286 (0.932–1.775)	0.126
**Marital status**												
Married	1045	73.7	Ref.		287	20.3	Ref.		287	20.3	Ref.	
Single/widowed/div.	334	80.9	0.860 (0.484–1.526)	0.605	83	20.1	0.822 (0.476–1.418)	0.481	83	20.1	0.819 (0.473–1.416)	0.474
**BMI (kg/m^2^)**			1.005 (0.966–1.045)				**0.951(0.915–0.989)**	**0.012**			**0.949(0.912–0.987)**	**0.009**
**Antihypertensive Treatment**												
No									42	9.3	Ref.	
Yes									328	23.8	**1.953 (1.307–2.918)**	**0.001**
A	262	84.8	Ref.		62	20.1	Ref.		62	20.1	Ref.	
B	251	83.1	1.096 (0.682–1.762)	0.704	65	21.5	1.421 (0.917–2.202)	0.116	65	21.5	1.418 (0.912–2.205)	0.121
C	281	92.1	**2.865 (1.612–5.095)**	**<0.001**	69	22.6	**1.641 (1.052–2.560)**	**0.029**	69	22.6	1.560 (0.995–2.445)	0.053
D	277	91.4	0.778 (0.436–1.389)	0.396	81	26.7	1.051 (0.607–1.819)	0.859	81	26.7	1.074 (0.618–1.865)	0.800
E	218	70.3	0.627 (0.393–1.000)	0.050	80	25.8	**2.231 (1.406–3.538)**	**0.001**	80	25.8	**2.401 (1.502–3.838)**	**<0.001**
F	90	29.9	**0.038 (0.021–0.068)**	**<0.001**	13	4.3	**0.184 (0.083–0.411)**	**<0.001**	13	4.3	**0.296 (0.127–0.691)**	**0.005**

R^2^: Coefficient of determination; BMI: Body Mass Index; The community health centres (CHCs) in cities A, B, C and D were Hospital-funded; the CHC in city E was Government-funded; the CHC in city F was privately-funded.

**Table 5:** Factors associated with antihypertensive drug treatment and optimal control of blood pressure (with financing system as a covariate).

	Treatment by antihypertensive drug (R^2^ = 0.382)				Optimal blood pressure control (R^2^ = 0.076)				Optimal blood pressure control with antihypertensive treatment as a covariate (R^2^ = 0.089)			
	n	%	Adjusted odds ratio (95% C.I.)	p	N	%	Adjusted odds ratio (95% C.I.)	p	n	%	Adjusted odds ratio(95% C.I.)	p
**Age (years)**			**1.032 (1.019–1.046)**	**<0.001**			1.011 (0.998–1.024)	0.090			1.009 (0.996–1.021)	0.190
**Gender**												
Male	633	71.2	Ref.	0.611	172	19.3	Ref.	0.477	172	19.3	Ref.	0.450
Female	746	79.3	0.927 (0.691–1.243)		198	21.0	1.102 (0.844–1.438)		198	21.0	1.109 (0.848–1.450)	
**Occupation**												
Employed	208	63.4	Ref.		63	19.2	Ref.		63	19.2	Ref.	
unemployed	451	74.2	0.952 (0.642–1.412)	0.808	123	20.2	1.099 (0.751–1.609)	0.625	123	20.2	1.106 (0.754–1.624)	0.606
Retired/in school/others	460	78.6	1.266 (0.841–1.907)	0.258	124	21.2	0.984 (0.673–1.439)	0.935	124	21.2	0.976 (0.665–1.432)	0.901
**Educational status**												
Primary or lower	626	80.1	Ref.		156	19.9	Ref.		156	19.9	Ref.	
Secondary	368	65.9	0.780 (0.561–1.085)	0.140	106	19.0	1.325 (0.967–1.817)	0.080	106	19.0	1.340 (0.974–1.842)	0.072
Tertiary or above	121	70.8	1.337 (0.754–2.371)	0.320	47	27.5	**2.555 (1.631–4.003)**	**<0.001**	47	27.5	**2.477 (1.577–3.892)**	**<0.001**
**Medical insurance**												
Absence	583	84.7	Ref.		135	19.6	Ref.		135	19.6	Ref.	
Presence	796	69.7	0.729 (0.509–1.044)	0.085	235	20.6	1.202 (0.883–1.635)	0.243	235	20.6	1.234 (0.905–1.682)	0.184
**Marital status**												
Married	1045	73.7	Ref.		287	20.3	Ref.		287	20.3	Ref.	
Single/widowed/div.	334	80.9	0.863 (0.488–1.525)	0.612	83	20.1	0.837 (0.486–1.444)	0.523	83	20.1	0.832 (0.481–1.439)	0.511
**BMI**			1.005 (0.966–1.045)	0.077			**0.951(0.915–0.988)**	**0.011**			**0.948 (0.912–0.986)**	**0.007**
**Antihypertensive Treatment**												
No									42	9.3	Ref.	
Yes									328	23.8	**2.001 (1.343–2.980)**	**0.001**
**Financing system**												
Hospital	1071	87.9	Ref.			22.7	Ref.		277	22.7	Ref.	
Government	218	70.3	**0.462 (0.325–0.656)**	**<0.001**		25.8	**1.628 (1.157–2.291)**	**0.005**	80	25.8	**1.784 (1.259–2.526)**	**0.001**
Private	90	29.9	**0.031 (0.019–0.052)**	**<0.001**		4.3	**0.146 (0.069–0.310)**	**<0.001**	13	4.3	**0.240 (0.107–0.535)**	**<0.001**

R^2^: Coefficient of determination; BMI: Body Mass Index.

When the regression model was re-constructed by classifying the CHCs according to the financing model with hospital-funded CHC as a reference (**Table 5**), the Government-funded (aOR 0.462, 95% C.I. 0.325–0.656, p<0.001) and private-funded CHCs (aOR 0.031, 95% C.I. 0.019–0.052, p<0.001) were associated with lower treatment rates. However, Government-funded CHCs were significantly more likely (aOR 1.628, 95% C.I. 1.157–2.291, p<0.001) and private-funded CHC less likely (aOR 0.146, 95% C.I. 0.069–0.310, p<0.001) to achieve optimal BP control than hospital-funded CHCs. When antihypertensive treatment was included as a covariate in the regression analysis with BP control as the outcome, similar findings on the comparisons of CHCs according to the financing models were observed (**Table 5**). These findings reflected significant differences in the general population among these communities, but implied that the regression analyses could control for these inter-city differences as the associated factors were similarly significant when models were constructed using different variables.

### Other Factors Associated with Blood Pressure Management

Older age was associated with prescriptions of antihypertensive treatment (AOR 1.035, 95% C.I. 1.022–1.049 per one mmHg increase of BP, p<0.001) and BP control (AOR 1.013, 95% C.I. 1.000–1.026, p = 0.043), but the latter association no longer exists when antihypertensive treatment was included as a covariate (**Table 4**). When compared with educational status at primary level or below, patients having secondary (AOR 1.475, 95% C.I. 1.058–2.058, p = 0.022) and tertiary level or above (AOR 3.101, 95% C.I. 1.906–5.044, p<0.001) were more likely to have optimal BP control (**Table 4**). In addition, people having higher body mass index were less likely to have their BP controlled (AOR 0.951, 95% C.I. 0.915–9.989, p = 0.012), irrespective of whether antihypertensive drug treatment was controlled. There was no collinearity detected among the covariates tested in the regression analyses.

## Discussion

### Major Findings

It was found that for treatment rates, hospital-funded CHCs were significantly higher than the Government-funded CHCs, followed by the privately-funded CHCs. For BP control rates, the Government-funded CHCs were the highest, whilst privately-funded CHCs had the lowest rate. Multivariate regression analysis also demonstrated that the privately-funded CHC had the poorest treatment and control rates of hypertension when potential confounders were controlled. This holds true whether antihypertensive treatment was controlled as a covariate in the regression analyses. Another factor associated with antihypertensive treatment included older age; while higher educational levels and lower BMI were associated with better BP control.

### Relationship to Literature and Explanation of Findings

From a recent survey among Tibetans hypertensive patients aged ≥40 years living at high altitude, 2.6% received antihypertensive treatment and only one patient out of 701 study participants had optimal BP control [20]. Another recent study involving more than 13,800 Southern Chinese aged ≥20 years found that the treatment and control rates were 37.9% and 13.5% in the urban regions, and were 10.4% and 3.4% in rural regions, respectively [21]. Even lower control rates have been reported among people in rural China (2%), urban India (12%) rural India (9%) [22]. The treatment and control rates of BP in Beijing China were found to be 35.9% and 11.5%, respectively [23]. Therefore the management of hypertension as reported in this study achieved better treatment and control rates than those evaluated by other studies. Since our participants were recruited from clinics instead of from the general population, it is not surprising to find these outcome measures as more optimal.

There are obvious differences among the three types of CHCs in terms of the source of financial support and the organizational structure. For the Government-funded CHCs, these supports include financial injections from the government annual budget which includes public health funding, initial establishment funding, equipment purchase, routine operating fees, staff costs and other forms of funding. Whist the fees charged from patients on clinical examination, medical treatment, drug sales, etc, which serve as the revenue of government-funded CHCs mostly goes to the local government. This particular feature renders the government-funded CHCs the greatest degree of a non-profit nature, which would reduce the possibility of excessive and unnecessary prescription of drugs to patients in order to maximize profits, as compared to hospital-funded CHCs and private-funded CHCs which have to be financially self-sufficient under the current market-orientated health system [24], [25]. Hospital- and private-funded CHCs in general receive a limited support on public health funding from the government. These CHCs often generate more profits largely from providing clinical medical services charged on a fee-for-service basis. In addition, whilst all CHCs were subject to industry supervision and monitoring from the Health Bureau to ensure the quality of services provided, the Government-funded CHCs had more connections with other Governmental agencies [26]. Also, studies found pharmaceutical companies had strong connections with secondary and tertiary hospitals, which are the holding hospitals of the hospital-managed CHCs [27], [28], and the physicians working in the out-patients clinics of hospital-funded CHCs were also used to working in secondary and tertiary sectors at the same time, where drug prescriptions are more common in management of chronic diseases than in the primary care sector. One might therefore speculate that physicians in hospital-funded CHCs could have lower threshold to prescribe antihypertensive drugs as their usual practice. Primary care providers in the Government-funded CHCs might not be as strongly incentivized than hospital-funded CHCs to prescribe antihypertensive agents during patient encounters where pharmacotherapy was not perceived as a must e.g. when elevation in blood pressure was only marginal which could be managed with educational and lifestyle modification counseling before drug treatment. The majority of profits made in Government-funded CHCs did not contribute to clinic income whereas a significant proportion of profit margins from consultations and prescriptions will go directly into Hospital- and privately-funded CHCs. Furthermore, physicians in privately-funded CHCs could be more hesitant to prescribe medications if not absolutely necessary as this involves extra payment from patients.

Nevertheless, greater proportions of patients in the Government-funded CHCs had optimal BP control, despite their lower treatment rates than the hospital-funded CHCs. One possibility is that under the government-funded CHC model, primary care service are provided directly from the local government, it leads to stronger and better policy implementation. The Government-funded CHCs creates a more robust chronic disease management infrastructure including the establishment of clinical guidelines and lifestyle modification initiatives [29]. The greater amount of funding for the Government CHCs could translate into more clinic-based programmes on self-management, like medication compliance-enhancing intervention and lifestyle modification initiatives. Further studies are warranted to explore whether there exists differences in counseling practices for patients with chronic diseases between CHCs with different financing modalities. Moreover, there is also stronger emphasis of the Government-funded CHCs on strengthening the role and responsibility of the Government in optimizing partnership infrastructure and coordinative care among different primary care professionals for service provision in the clinic, hence explaining its superior performance in BP control.

Turning to the relationships between optimal BP control and demographics like age, educational levels, BMI, the current literature was mixed [23], [30]–[32]. A recent study found that no significant association was found with education and BMI for both men and women, but women in the age groups 50 years and older were significantly more likely to have controlled hypertension [30]. A study conducted in Beijing, China demonstrated a clear relationship between poor hypertension control with older age, lower educational attainment and central obesity [23]. The explanations of these associations have been extensively discussed elsewhere [23], [30]–[32].

### Strengths and Limitations

This is the first study which directly compared the healthcare financing systems in the PRD region by adopting a random sampling methodology in the selection of CHCs. However, some limitations should be mentioned. Firstly, we recruited only one CHC from each city and the sample size is modest, although the sampling was conducted in a systematic manner using random sampling methodology. In addition, we have included the management of one chronic condition as the outcome indicator for this comparison study, and the performance of other aspects such as preventive services, treatment of acute conditions, patients’ quality of life and longer-term health outcomes have not been evaluated. Also, we excluded patients who lived in their respective districts for less than one year. Since it is likely that a significant proportion of these more mobile residents were migrants, the generalizability of our study findings to them might be limited. Moreover, there are other factors apart from the healthcare system which could influence blood pressure control. These factors might be patient-related, such as dietary or exercise habits, smoking, alcohol drinking the severity of hypertension, and specific cultural attitudes to management of hypertension as a disease entity. Clinic- or physician-related factors include accessibility to healthcare, the time since the diagnosis of hypertension, the availability of different types of antihypertensive medications in the practice, the size of the CHCs, the number of clinic staff and the presence of treatment guidelines. The differences in proportion of migrants, which represent a more underprivileged group, could also be contributory. These factors have not been controlled in our regression models. Finally, ascertainment bias of blood pressure measurement among these cities should be addressed.

### Implications for Policy-making and Future Perspectives

We concluded that the privately-funded CHCs attained poorest healthcare outcomes when compared with other CHCs in this study but there exists factors other than the financing system which might confound the outcome we measured. The reasons of the poorer performance among the privately funded CHCs will require further exploration, including evaluation of components within the private financing model which might lead to poorer patient management. On the contrary, the strengths of the hospital and Government-funded system will need to be explored, including the quality of supervision and quality of care in addition to resources available to the different types of CHCs. Another implication is that the clinical guidelines and better lifestyle modification initiatives seen in Government-funded CHCs could be potentially replicated in the Hospital- and privately-funded CHCs. Furthermore, comparisons of these financing systems using other tracer markers could further inform policy-makers on future healthcare policy-making and health service implementation. For healthcare reforms involving health system design, it is recommended that the policy-makers analyze the essential key success factors which could lead to better health outcomes.

## References

[1] World Health Organization (1988) From Alma-Ata to the year 2000. Reflections at the midpoint. Geneva.

[2] Macinko J, Starfield B, Shi L (2003) The contribution of primary care systems to health outcomes within Organization for Economic Cooperation and Development (OECD) countries, 1970–1998. Health Serv Res. 38: 831–65. 10.1111/1475-6773.00149 PMC1360919 12822915

[3] Starfield B, Shi L, Macinko J (2005) Contribution of primary care to health systems and health. Milbank Q 83: 457–502. 16202000 10.1111/j.1468-0009.2005.00409.x PMC2690145

[4] Starfield B (2008) The future of primary care: refocusing the system. N Engl J Med. 13;359: 2087, 2091. 10.1056/NEJMp0805763 19005193

[5] World health report 2008 – primary health care: now more than ever. Geneva: World Health Organization, 2008.

[6] Meads G, Wild A, Griffiths F, Iwami M, Moore P (2006) The management of new primary care organizations: an international perspective. Health Serv Manage Res 19: 166–173. 16848957 10.1258/095148406777888125

[7] WHO Regional Committee for Europe resolution EUR/RC55/R8 on strengthening health systems as a continuation of the WHO Regional Office for Europe’s Country Strategy “Matching services to new needs”. Copenhagen, WHO Regional Office for Europe (2005)

[8] Rasella D, Aquino R, Barreto ML (2010) Impact of the Family Health Program on the quality of vital information and reduction of child unattended deaths in Brazil: an ecological longitudinal study. BMC Public Health. 29 10: 380. 10.1186/1471-2458-10-380 PMC3091549 20587036

[9] Improving Primary Health Care for All Australians. Commonwealth of Australia 2011. Available: http://www.healthissuescentre.org.au/documents/items/2011/02/363952-upload-00001.pdf. Accessed 2012 Jun 15.

[10] Eleventh Five Year Plan (2007–2012) Social Sector. Volume 2. Planning Commission, Government of India. Oxford University Press. New Delhi (2008)

[11] “Opinions on Deepening Pharmaceutical and Healthcare System Reform”. State Council. No.9 document. Mar 17, 2009. Available: http://www.gov.cn/jrzg/2009-04/06/content_1278721.htm. Accessed 2012 Jun 15.

[12] 2010 National Census. 2010 Main Social and Economic Indicators. Statistics Bureau of Guangdong Province Government. Published in May 2010.

[13] Gross Domestic Product index in Pearl River Delta 2009. Available: http://www.gdprice.org/2010/0128/1917.html. Accessed 2012 Mar 16.

[14] Accelerating the community health service development in Dongguan. No. 96 document.: Dongguan government (2007)

[15] Scheme on improving community healthcare management in Shenzhen.: Shenzhen government (2010)

[16] Guildelines on healthcare facilities management in sub-urban areas. No. 63 document.: Guangdong government (2008)

[17] Provisions on management of communith health service organizations in Xiangzhou district. No.107 document.: Zhuhai government (2002)

[18] Opinions on tackling the task on achieving social security. No. 14 document. Guangdong government (2007)

[19] Implementation guidance on the establishment of basic medical insurance system for urban residents. No. 75 document. Guangdong government (2007)

[20] McGlynn EA, Asch SM (1998) Developing a clinical performance measure. Am J Prev Med 14: 14–21. 9566932 10.1016/s0749-3797(97)00032-9

[21] Guthrie B. Inkster M. Fahey T (2007) Tackling therapeutic inertia: role of treatment data in quality indicators. BMJ 335: 542–4. 17855323 10.1136/bmj.39259.400069.AD PMC1976517

[22] Lawrence M (1997) Indicators of quality in health care. Eur J Gen Pract 3: 103–108.

[23] Chobanian AV, Bakris GL, Black HR, Cushman WC, Green LA, et al (2003) Seventh report of the Joint National Committee on Prevention, Detection, Evaluation, and Treatment of High Blood Pressure. Hypertension 42: 1206–1252. 14656957 10.1161/01.HYP.0000107251.49515.c2

[24] Hsiao WCL, Liu YL (1996) Economic reform and health - Lessons from China. New Engl J Med 335: 430–32. 8663878 10.1056/NEJM199608083350611

[25] Chen Z (2009) Launch of the health-care reform plan in China. Lancet 373: 1322–4. 19376436 10.1016/S0140-6736(09)60753-4

[26] Zhao DY, Liang H (2011) Role and function of government in the community health services development: Shanghai experience. Fudan Journal (Social Sciences) 3: 88–96.

[27] TriPoint Global Research. Available: http://chinapharmaceuticalsinc.com/CFMI_Report_070910_final.pdf. Published on 9 July 2010.

[28] Changing Landscape of China’s Pharmaceutical Distribution Industry. Booz & Company Inc. Available: http://www.booz.com/media/file/Changing_Landscape_of_China’s_Pharmaceutical_Distribution_EN.pdf. Published on 2012 Apr 8.

[29] Recent major strategic plan for pharmaceutical and healthcare system reform in Dongguan (2009–2011) No. 71 document: Dongguan government (2010)

[30] Zhao X, Li S, Ba S, He F, Li N, et al. (2012) Prevalence, Awareness, Treatment, and Control of Hypertension Among Herdsmen Living at 4,300 m in Tibet. Am J Hypertens. doi: 10.1038/ajh.2012.9. [Epub ahead of print]. 10.1038/ajh.2012.9 22357415

[31] Ma WJ, Tang JL, Zhang YH, Xu YJ, Lin JY, et al. (2012) Hypertension Prevalence, Awareness, Treatment, Control, and Associated Factors in Adults in Southern China. Am J Hypertens. doi: 10.1038/ajh.2012.11 [Epub ahead of print]. 10.1038/ajh.2012.11 22337206

[32] Prince MJ, Ebrahim S, Acosta D, Ferri CP, Guerra M, et al (2012) Hypertension prevalence, awareness, treatment and control among older people in Latin America, India and China: a 10/66 cross-sectional population-based survey. J Hypertens. 30: 177–87. 10.1097/HJH.0b013e32834d9eda 22134385

